# Experiences With the Levonorgestrel Intrauterine System Among Clients, Providers, and Key Opinion Leaders: A Mixed-Methods Study in Nigeria

**DOI:** 10.9745/GHSP-D-18-00242

**Published:** 2018-12-27

**Authors:** Gillian Eva, Geeta Nanda, Kate Rademacher, Anna Mackay, Omaye Negedu, Anne Taiwo, Leila Dal Santo, Mariya Saleh, Lucky Palmer, Tracey Brett

**Affiliations:** a Marie Stopes International, Washington, DC, USA.; b FHI 360, Washington, DC, USA.; c FHI 360, Durham, NC, USA.; d Marie Stopes International, New York, NY, USA.; e Marie Stopes International Organisation Nigeria, Abuja, Nigeria.; f FHI 360, Abuja, Nigeria.; g FHI 360, Johannesburg, South Africa.

## Abstract

**Background:**

The levonorgestrel intrauterine system (LNG IUS) is one of the most effective contraceptive methods, and it has noncontraceptive health benefits, including treatment for women with heavy menstrual bleeding. In 2016, Marie Stopes International Organisation Nigeria (MSION) expanded LNG IUS provision through training and support to 9 mobile outreach teams, 105 social franchise clinics, and 20 public-sector providers in 17 states. Information about the LNG IUS was added to awareness-raising materials, and community mobilizers provided information on the LNG IUS alongside other voluntary family planning methods.

**Methods:**

In 2016, Marie Stopes International, MSION, and FHI 360 examined clients' and providers' experiences with the LNG IUS to assess the potential for further scale-up of the method as part of a comprehensive approach to family planning in Nigeria. A mixed-methods approach was used including analysis of routine service data, supplemental data specific to LNG IUS clients, and in-depth interviews with LNG IUS clients, providers, and key opinion leaders.

**Results:**

Just under 1,000 LNG IUS were inserted from September 2016 to December 2017 in 16 states in channels supported by MSION, representing 0.4% of all long-acting and reversible contraceptive (LARC) services provided by the participating providers during this time frame. The vast majority (82%) of LARCs provided were implants. A small pool of providers was responsible for providing almost half of the LNG IUS services. Common reasons for women choosing the LNG IUS were reduced menstrual bleeding (61%), long-acting duration (52%), effectiveness (49%), and discreetness (42%). Almost 80% of the users first heard about the method from a provider. Almost all users and providers reported positive experiences with the method, noting the noncontraceptive benefits and fewer side effects compared with other methods. All providers who were interviewed said they would continue offering the LNG IUS. Several key opinion leaders mentioned a total market approach incorporating both public and private sectors would be needed to successfully scale up the LNG IUS.

**Conclusion:**

Reduced menstrual bleeding and fewer side effects compared with other methods were identified as important attributes of the LNG IUS by clients, providers, and key opinion leaders. Challenges to uptake of the LNG IUS include difficulty with introducing a new method within a busy service delivery infrastructure and limited awareness and demand-generation activities on the LNG IUS specifically. A comprehensive product introduction approach with coordinated demand- and supply-side activities may be required for this method to reach its full potential.

## Abstract (precis)

Between September 2016 and December 2017, Marie Stopes International Organisation Nigeria introduced the LNG IUS in 16 Nigerian states to increase method choice. Just under 1,000 devices were inserted, representing less than 1% of all long-acting reversible contraceptives provided. Qualitative feedback from opinion leaders, providers, and LNG IUS users found important benefits to users and suggested coordinated demand- and supply-side activities, including user champions and supportive providers to generate interest in the method, would be needed for successful scale-up.

## BACKGROUND

The levonorgestrel intrauterine system (LNG IUS), a long-acting and reversible contraceptive (LARC) method, is one of the most effective forms of contraception available and has important noncontraceptive health benefits, such as treatment for heavy menstrual bleeding and potential alleviation of anemia in some women. Possible side effects include acne, headaches, breast tenderness, and weight gain.^1^^,^^2^ First introduced nearly 30 years ago, the LNG IUS has been popular in countries where it is available, but access to the method in low- and middle-income countries (LMICs) has been very limited, largely due to the current high price of the product.^3^^,^^4^ New, more affordable LNG IUS products are starting to become available globally, and early introduction efforts are underway in several LMICs,^5^ including Nigeria. Introduction of new contraceptive methods has the potential to increase voluntary contraceptive use^6^ and gives women and men a better chance of finding a method that suits them.

As part of the global Family Planning 2020 (FP2020) initiative launched in 2012, the government of Nigeria has committed to reducing unmet need for family planning and increasing modern contraceptive prevalence to 27% among all women by 2020,^7^ compared with a prevalence of 11% in 2013.^8^ Data from the 2013 Nigeria Demographic and Health Survey indicated that 13% of all women (ages 15 to 49) in Nigeria had an unmet need for family planning.^8^ Family planning use varies widely across states, with the modern contraceptive prevalence rate ranging from 5.9% in Kano to 23.3% in Lagos, according to 2017 Performance Monitoring and Accountability 2020 (PMA2020) data.^9^ Recent targeted initiatives in both the public and private sectors have led to increased voluntary uptake of LARCs (particularly implants) in Nigeria, including among lower-income women and women living in the more rural and conservative northern states^10^ who often have limited or no access to family planning.

According to the 2013 Nigeria Demographic and Health Survey, the copper intrauterine device (IUD) comprised a small portion (5%) of Nigeria's overall contraceptive method mix,^8^ and use of the LNG IUS is too low to be included as a separate method in national surveys. Mirena, the 5-year LNG IUS product distributed by Bayer HealthCare Pharmaceuticals Inc., is available on a limited scale in the commercial sector, at a cost to clients (including insertion) ranging from US$90 to US$275 in private clinics in Abuja, according to a recent assessment.^11^ Several other LNG IUS products are starting to be introduced in Nigeria, including Emily (HLL Lifecare Ltd.), Eloira (Pregna International Ltd.), and AVIBELA (Medicines360) (information is from personal communications with respective suppliers of LNG IUS products, January 2018). In addition to these commercial products, a free, non-branded LNG IUS product is donated by the International Contraceptive Access (ICA) Foundation, a public-private partnership between Bayer HealthCare Pharmaceuticals Inc. and the Population Council. This product is being offered by several organizations across the country including Marie Stopes International Organisation Nigeria (MSION).

Small-scale LNG IUS introduction pilots led by several service delivery groups have been underway for some time in Nigeria. However, to our knowledge, this is the first formal research to be conducted about the experiences of LNG IUS users and providers within the country. Using evidence from MSION's LNG IUS program, this article aims to explore the perceptions and experiences of early adopters of the LNG IUS, LNG IUS providers, and key opinion leaders. The results identify both challenges and opportunities with method introduction and can help inform further introduction and scale-up of the method in Nigeria and beyond.

## PROGRAM DESCRIPTION

MSION supports high-quality family planning counseling and a range of voluntary contraceptive services, including LARCs and permanent methods, through a network of delivery points across 33 states in Nigeria. In underserved rural areas, MSION expands the contraceptive choices available in public facilities by offering voluntary LARCs and permanent methods through 12 mobile outreach teams and by training public-sector providers in LARC provision, while in peri-urban areas MSION supports the BlueStar network of 253 social franchise clinics (usually small, private clinics staffed by a mid-level provider). In 2017, MSION provided more than 2 million voluntary family planning services (not including condoms or emergency contraception), of which almost 1 million were voluntary LARC services.

The ICA Foundation donated 1,400 LNG IUS units to MSION between 2010 and 2014 (these were programmed through routine service delivery) and a further 3,000 units in 2016–2017. With this additional donation and with funding from the United States Agency for International Development (USAID) in 2016, MSION expanded LNG IUS provision through training and support to 9 mobile outreach teams, 105 social franchise clinics, and 20 public-sector providers. This period of expanded provision in 2016–2017 is referred to in this article as “the program.” Activities under the program included training 136 providers and 12 MSION supervisors on LNG IUS provision, generating awareness of the method among potential users via outreach by community mobilizers, and monitoring of service provision. Providers from 17 states across Nigeria were trained, with LNG IUS services ultimately delivered in 16 states. Social franchise clinics were selected that had high volumes of family planning services and an experienced family planning provider trained in provision of LARCs including IUDs, covering 13 states (Abia, Anambra, Benue, Cross River, Delta, Ebonyi, Edo, Enugu, Imo, Lagos, Ogun, Ondo, and Oyo). Nine mobile outreach teams were included, covering 7 states (Abuja in the Federal Capital Territory, Benue, Cross River, Gombe, Lagos, Osun, and Sokoto). Public-sector providers were trained in 5 states (Benue, Cross River, Ogun, Ondo, and Oyo). Marie Stopes International (MSI) and MSION partnered with FHI 360 to examine experiences with provision and use of the LNG IUS in these settings among both clients and providers during the program period, and to assess the potential for further scale-up of the method more broadly.

Marie Stopes International Nigeria expanded LNG IUS provision in 2016 through training and support to mobile outreach teams, social franchise clinics, and public-sector providers.

The providers selected for involvement in the program included a mix of nurses, midwives, and doctors. They took part in a 3-day training between April and November 2016 that covered history taking, general family planning counseling, steps on infection prevention, and demonstration of insertion and removal of the LNG IUS using models and practicing with clients who wanted the LNG IUS. Trainers or supervisors observed all providers inserting LNG IUS with clients, either during the training or during a follow-up observation in a facility, before reaching competency as determined by MSI global clinical standards. Technical competence was monitored during routine clinical follow-up visits every 2 months.

Following the training, the LNG IUS was introduced alongside other family planning methods through mobile outreach teams, social franchise clinics, and public-sector providers supported by MSION. As with other contraceptive services, there was no charge for the LNG IUS provided through mobile outreach or at public-sector facilities. Social franchise clinics charged a consultation fee of 2,000 to 3,000 Naira (US$6 to $8) for LNG IUS services during the first year of the program, which was reduced to 1,500 Naira (US$4) in October 2017 to make the cost of the LNG IUS comparable with implant and copper IUD services, which range from 1,000 to 2,000 Naira (US$3 to $6).

Information about the LNG IUS was added to existing awareness-raising materials, including flip charts and posters. In addition, community mobilizers already providing family planning information at the community level were trained to provide information about the LNG IUS, including duration of use, side effects, risks including conditions that might render the use of the method inadvisable, and effectiveness, as well as the noncontraceptive benefits of the method.

Providers received a small number of products once they achieved competency and could request more as needed, with additional supplies delivered by MSION within 1 week of the provider's request. There were no reported LNG IUS stock-outs during the program. Since the end of the program in December 2017, MSION's trained providers continue to offer donated LNG IUS as part of a broader method mix.

## METHODS

We used a mixed-methods approach to evaluate experiences with and attitudes toward the LNG IUS, drawing on different data sources, including routine MSION service data, supplemental data specific to LNG IUS clients, and qualitative in-depth interviews with LNG IUS clients, providers, and key opinion leaders (Table 1). We also included results from MSION's 2017 client exit interviews for comparative purposes.

**TABLE 1.** Data Sources

Data Source	Frequency	Participants
Routine MSION service data	Collected by providers at time of service on ongoing basis	All MSION family planning clients
Supplementary LNG IUS client data	Collected by providers at time of service as part of LNG IUS program	Sample of LNG IUS clients (N=388)
MSION client exit interview data^a^	Collected by MSION annually	Sample of MSION LARC clients (N=692)
In-depth interviews	Conducted in 2017	LNG IUS clients (N=33) LNG IUS providers (N=32) Key opinion leaders (N=17)

Abbreviations: LARC, long-acting reversible contraception; LNG IUS, levonorgestrel intrauterine system; MSION, Marie Stopes International Organisation Nigeria.

a Used for comparison purposes, not part of LNG IUS study protocol.

Ethical approval was received from the Nigerian Institute of Medical Research, the MSI independent Ethics Review Committee, and FHI 360's Office of International Research Ethics.

### Routine MSION Service Data and Supplemental LNG IUS Client Data

The research team used routine MSION service data on the number of family planning services delivered, which MSION collected throughout the program via paper forms at all facilities involved in LNG IUS introduction. We also collected supplemental data from LNG IUS clients at the time of service, through 4 mobile outreach teams and at 47 social franchise clinics, using paper-based questionnaires. The questionnaires included questions about women's reasons for choosing the LNG IUS, how they heard about the method, what method they would have chosen if the LNG IUS had not been available that day, and sociodemographic characteristics (age, parity, education level, and marital status).

MSION conducted client exit interviews with a sample of MSION clients after they received a service from MSION between November 8 and November 29, 2017, in order to capture sociodemographic characteristics. Client exit interviews are part of MSION's routine monitoring and evaluation activities and were not part of the study protocol; as such, they were not necessarily conducted at the sites where LNG IUS services were offered. We have included them to provide broad comparisons among LNG IUS users and other MSION LARC clients. For outreach, we conducted the client exit interviews in the same states as the LNG IUS rollout, plus Kano state. For social franchise, they were conducted in the same states as the LNG IUS rollout, excluding Anambra, Ebonyi, and Imo states.

Our indicators of interest included the following: sociodemographic characteristics of LNG IUS users, why women chose to use it, what alternative method they would have used, and how they initially found out about the LNG IUS. Where appropriate, we explored differences by service delivery channel and with other LARC users, using chi-square (χ2) tests or *t* tests as applicable. Descriptive data analysis was conducted using SPSS version 22,^12^ using univariate and bivariate analysis.

### Qualitative Interviews With LNG IUS Clients and Providers

Between March and October 2017, we interviewed LNG IUS clients and providers to document their experiences using and providing the LNG IUS, respectively, attitudes toward the method including advantages and disadvantages, and potential considerations for expanding access to the method. Semistructured interview guides were used for all interviews.

A sub-sample of LNG IUS clients who had given permission to be contacted at the time of service was invited to participate in an in-depth interview 3 months after the clients received the LNG IUS. Interviews were conducted in person at a health facility convenient to the woman, in English and other local languages, based on the respondent's preference. We initially aimed to reach 40 to 50 women from across the delivery channels, estimating that these numbers would be sufficient to identify major themes^13^ and take into account feasibility constraints related to financial and time considerations. Participants were recruited via convenience sampling. Ultimately, due to time constraints and challenges reaching women, we interviewed a total of 33 women.

Providers who were trained on provision of the LNG IUS and had provided the method in one of the participating facilities were similarly recruited via convenience sampling, with the aim of reaching 20 to 30 providers, assuming these numbers would be sufficient to identify major themes.^8^ Interviews were conducted in English at the provider's facility. We interviewed 32 providers.

We obtained informed consent from all participants before initiating the in-depth interviews. LNG IUS clients were compensated 2,000 Naira (US$6) for transportation costs. Providers did not receive compensation for participation. We audio-recorded all in-depth interviews (except for 2 client interviews and 1 provider interview in Edo state that were documented with detailed notes) and transcribed them, translating into English when applicable. We analyzed the in-depth interview data thematically using NVivo version 11,^14^ first coding the data according to a framework derived from themes in the interview guide and emerging themes from the interview data, then developing memos related to the larger themes and emerging sub-themes.

### Qualitative Interviews With Key Opinion Leaders

Between January and September 2017, we conducted interviews with key opinion leaders to document their perceptions of the LNG IUS and attitudes about opportunities and challenges associated with further introduction and scale-up of the LNG IUS more broadly within Nigeria. Key opinion leaders were identified by local FHI 360 and MSION staff based on their experience and expertise in the field of reproductive health in Nigeria and, in some cases, with LNG IUS provision. Seventeen interviews were conducted with individuals including representatives from government institutions, NGOs, academic institutions, donor groups, and procurement organizations. Opinion leaders who were interviewed did not receive compensation. All interviews were conducted in English either in person (n=15) or by phone (n=2). Four respondents declined to be audio-recorded; in these cases, detailed written notes of the interviews were taken. In all other cases, interviews were audio-recorded and transcribed. Results were analyzed thematically in Microsoft Excel by categorizing responses according to a framework based on themes from the interview guide.

## RESULTS

Between September 2016 and December 2017, the 9 trained mobile outreach teams, 76 of the 105 trained social franchise providers, and 7 of the 20 trained public-sector providers delivered 990 LNG IUS insertions. Almost all clients received LNG IUS services at either a social franchise clinic (53.0%, n=525) or through mobile outreach (44.5%, n=441). A small number received the method through participating public-sector facilities (2.4%, n=24); these cases are not included in the following analysis. The rate of voluntary uptake of the method was fairly consistent throughout the program period.

Most (83%, n=95) of the participating providers delivered fewer than 10 LNG IUS services during the 16-month program, while 9 providers (8%) (4 mobile outreach teams and 5 social franchise clinics) provided on average 58 LNG IUS services each (ranging between 24 and 89). These 9 providers delivered just over half (52.5%) of all LNG IUS insertion services.

Most of the 115 providers in the program delivered fewer than 10 LNG IUS services, while 9 providers delivered just over half of all LNG IUS insertion services.

LNG IUS services represented 0.4% of all voluntary LARC services provided by the participating mobile outreach teams and social franchise clinics that provided at least 1 LNG IUS service during this time frame: 0.7% among the social franchise clinics and 0.3% among the mobile outreach teams (Table 2). The majority of LARCs provided were implants (82.3%)—88.2% of LARCs provided in mobile outreach settings were implants and 69.4% of LARCs provided in social franchises were implants.

**TABLE 2.** Voluntary LARC Provision in Facilities Participating in LNG IUS Introduction Program, Nigeria, September 2016–December 2017

	Social Franchise No. (%)	Mobile Outreach No. (%)	Total No. (%)
Implants	54,720 (69.4)	151,943 (88.2)	206,663 (82.3)
IUDs	23,649 (30.0)	19,889 (11.5)	43,538 (17.3)
LNG IUS	525 (0.7)	441 (0.3)	966 (0.4)^a^
Total	78,894 (100)	172,273 (100)	251,167 (100)

Abbreviations: IUDs, intrauterine devices; LARC, long-acting reversible contraception; LNG IUS, levonorgestrel intrauterine system.

a Public-sector figures on provision of other LARCs were not available so the 24 LNG IUS provided through public-sector facilities are not presented in this table.

### LNG IUS Client Sociodemographic Characteristics

Sociodemographic data were available for 29.6% of LNG IUS clients (n=286), although some of these women did not answer all of the questions related to sociodemographic characteristics.

According to this sub-sample, the mean age for LNG IUS clients was 34.0 years (standard deviation [SD]=6.5). Almost half (47.2%; n=134) of LNG IUS clients in the sample were 35 years or older; 69.5% (n=146) of LNG IUS clients had completed secondary education or higher. The vast majority of LNG IUS clients in the sub-sample were married (93.8%; n=259), and these LNG IUS clients had on average 4.4 children (SD=2.6) (n=272).

MSION's 2017 client exit interviews were conducted with 692 LARC clients (91 IUD clients and 601 implant clients). To provide a broad comparison point, we compared client exit interview data with the service delivery data collected for LNG IUS users, and we found that the profile of the clients choosing LNG IUS (for whom we have sociodemographic data) differed in a number of ways from MSION's average LARC client profile. LNG IUS clients were more highly educated (29.5% of LNG IUS clients had education beyond secondary school, compared with 7.6% of MSION LARC clients [χ2=44.7, degrees of freedom (*df*)=3, *P*=.000]); older (mean age of LNG IUS clients was 34 years, compared with 30 years for MSION LARC clients [*t*=9.031, *df*=960, *P*<.001]); and had more children (4.4 on average compared with 3.8 for MSION LARC clients [*t*=3.401, *df*=958, *P*=.001]). The vast majority of all clients were married, but LNG IUS clients were more likely to be married (93.8% of LNG IUS clients were married compared with 89.1% of MSION LARC clients [χ2=5.103, *df*=1, *P* =.02]).

### Supplemental LNG IUS Client Data

Due to challenges in getting all providers to systematically collect the supplemental data specific to LNG IUS clients, these data were collected from only 38% (n=400) of LNG IUS clients during the program period—from 47 social franchise clinics (285 clients), 4 mobile outreach teams (103 clients), and 3 public-sector providers (12 clients). The 12 clients from the public sector were not included in the analysis, leaving a sample of 388 cases in the analysis.

#### Reasons for Choosing the LNG IUS

LNG IUS clients were asked, after they had chosen the method, why they chose the LNG IUS (Table 3). Multiple responses were possible and responses were offered spontaneously.

**TABLE 3.** Reasons for Choosing the LNG IUS (N=326)^a^

Reason^a^	No. (%)
Reduced menstrual bleeding	197 (61.4)
It lasts for a long time	167 (52.0)
Effective	157 (48.9)
Nobody will know	134 (41.7)
It is convenient/don't need to do anything	100 (31.3)
Won't affect future fertility	98 (30.7)
Few side effects	86 (26.9)
Recommended by friend or family	63 (19.7)
Don't want more children	59 (18.5)
Want to delay pregnancy for at least 2 years	54 (17.0)
Can use while breastfeeding	51 (16.0)
Affordable here	39 (12.3)
Other	11 (3.3)
Not sure	3 (0.9)

Abbreviation: LNG IUS, levonorgestrel intrauterine system.

a Source: Supplementary LNG IUS client data collected at time of service. 326 women gave at least 1 response to this question; the women could provide multiple responses and responses were offered spontaneously.

One of the most cited reasons for choosing the LNG IUS at time of service delivery was the potential of reduced menstrual bleeding.

The most commonly mentioned reasons for choosing the LNG IUS were: reduced menstrual bleeding (61.4%); long duration of effectiveness (“it lasts for a long time”) (52.0%); effectiveness (48.9%); and discreetness (“nobody will know”) (41.7%). Reduced menstrual bleeding was mentioned by 91.3% of mobile outreach clients but only 51.5% of social franchise clients.

#### Method of Choice if the LNG IUS Were Unavailable

Women who chose the LNG IUS were asked what method, if any, they would have chosen if the LNG IUS had not been available (data not shown). The majority of clients who gave a response to this question (n=332) would have chosen another LARC (78.9%, n=262): either a copper IUD (49.7%, n=165) or an implant (29.2%, n=97).

Thirteen percent of clients (n=43) would have chosen a short-acting method and 3.9% (n=13) would have chosen a traditional method. Only 2 clients (0.6%) stated that they would have gone elsewhere for an LNG IUS, and 10 (3.0%) reported that they would not have used any method if the LNG IUS were not available.

#### Initial Source of Information About the LNG IUS

In a multiple-response question on the source of clients' information about the LNG IUS, with answers given spontaneously, most clients who responded to this question (n=347) mentioned first hearing about the LNG IUS from a health care provider, either on the day of service (54.4%), on another day (14.1%), or through a referral from another health care provider (11.0%) (Table 4). Mobile outreach clients were more likely to have learned about the method from clinic staff on the day they received the method (96.0%) versus social franchise clients (36.4%) who received LNG IUS information through more diverse sources—37.6% of social franchise clients heard about the method from a friend or family member, and 15.1% heard about it from a community health worker (CHW) or volunteer.

**TABLE 4.** First Source of Information About the LNG IUS (N=347)^a^

First Source of Information	No. (%)
From clinic staff today	189 (54.4)
Friend/family member	97 (28.0)
From clinic staff on another day	49 (14.1)
Community health worker/volunteer	40 (11.5)
Referred by other health provider	38 (11.0)
Poster/flyer	15 (4.3)
Other	7 (2.0)

Abbreviation: LNG IUS, levonorgestrel intrauterine system.

a Source: Supplementary LNG IUS client data collected at time of service. 347 women gave at least 1 response to this question; the women could provide multiple responses and responses were offered spontaneously.

### Qualitative Interviews With LNG IUS Clients and Providers

We interviewed 33 women (24 from social franchise clinics; 2 from public-sector facilities; 7 through mobile outreach teams), 30 of whom were currently using the LNG IUS and had been for at least 3 months. Three had recently discontinued use. Sociodemographic information about the in-depth interview participants is shown in Table 5. We also interviewed 32 health care providers (28 female and 4 male) who were mostly nurses and midwives. Data on LNG IUS insertions were available for 24 providers. Among these providers, the number of LNG IUS insertions ranged from 1 to 40, with an average of 7 insertions.

**TABLE 5.** Sociodemographic Characteristics and Family Planning Use of LNG IUS Clients (N=33)^a^

Sociodemographic Characteristics	Women
**Age, years, mean**	33.8
**Marital status, No.**	
Married	27
Not married	2
**Number of children, mean**	3.2
**Education, No.**	
None/some or completed primary	9
Some or completed secondary	12
Post-secondary	8
**Currently using the LNG IUS, No.**	30
**Recently removed the LNG IUS, No.**	3
**Previously used family planning, No.**	24
**Family planning methods ever used, No.b**	
Condoms	5
Pills	5
Injectables	14
Implants	8
Copper IUD	8
Other	2
**Desire for future children, No.**	
Yes	17
No	9
Maybe	3

Abbreviations: IUD, intrauterine device; LNG IUS, levonorgestrel intrauterine system.

a Source: In-depth interviews with LNG IUS clients. Sociodemographic data and data on desire for future children are missing for 4 women.

b Not mutually exclusive.

#### Women's Reasons for Choosing the LNG IUS

The most common reason given for choosing the LNG IUS was that a health care provider recommended it.

*They said it will work well with my body. I might forget to take the pills and I don't like injection. So that's why I prefer it.* —User, age 28, 2 children

Some women also chose the LNG IUS because they wanted a method that could help with heavy bleeding or fibroids.

*From what she [the provider] said, I prefer it, because it will control the bleeding and it also reduces the date [length of bleeding], because I told her, I have heavy flow for 7 days and she said it will reduce the flow.* —User, age 30, 3 children

*She actually told me that it reduces fibroid … I was diagnosed with multiple fibroids and I've been afraid of undergoing the surgery … so I started using this herbal method, it wasn't working, and then she [the provider] told me about this method, that it reduces fibroids, so that was why I had it inserted.* —User, age 37, 1 child

Some participants also chose the LNG IUS because they had negative experiences with other contraceptive methods.

*I had taken [the] injectable for 6 months but blood was coming out and it didn't stop at all. That's the reason I chose another one [type of method].* —User, age 34, 3 children

#### Providers' LNG IUS Insertion Experiences

All providers expressed confidence in their current ability to insert the LNG IUS; however, 13 of the 32 providers mentioned experiencing challenges, typically during their first insertion. Challenges included difficulty using the insertor, difficulty inserting in women with fibroids, forgetting to pull back the string, difficulty inserting in women with a small or anteverted uterus, and difficulty inserting when patients were not menstruating (because the cervix was perceived as being too tight). One provider indicated that she had carried out a total of 5 insertions:

*I had a challenge with some that had a huge uterine fibroid … the LNG IUS couldn't sit in there so I had to send her for scanning … she ended up taking Jadelle [implants]. I wouldn't want to insert something that I wasn't confident of where I wanted it to be.* —Provider, social franchise, Edo

#### Perceptions of the LNG IUS Among Clients and Providers

In general, method satisfaction was high among the current users interviewed, with all 30 saying they intended to continue using the method, and 28 stating they would recommend the LNG IUS method to their friends—7 of whom had already done so.

Satisfaction was high among LNG IUS users, with all 30 who were interviewed saying they intended to continue using the method.

*I think after this one I will still insert it again, and I also encourage some other women to go for it, especially those that are having problems with other methods, I encourage them to go for this one.* —User, age 43, 6 children

When asked about perceived advantages with LNG IUS use, women most often mentioned that they liked not experiencing any side effects while using the method as compared with other methods. “It works well for my body” was a sentiment frequently cited by users.

*I'm comfortable, I like it, it does not give me any problem, not any health problem that some women will say—head pain, back pain, leg pain—all these things, I did not experience anything.* —User, age 43, 6 children

In some cases, women were also happy that the method regulated or reduced their menstrual bleeding and that they did not experience any pain when using the method:

*I am very okay with it. There is no excessive bleeding compared to the last one. It did just like they told us. I am satisfied 100%.* —User, age 52, 4 children

Three women specifically cited the discreet nature of the method as a perceived advantage. Nine users had something negative to say about the method, including that they did not like the initial spotting that occurred post-insertion, being able to feel the string, and having irregular menstruation or amenorrhea.

Of the 3 women who had discontinued use, 2 were due to involuntary expulsion and the third was not satisfied with the method's side effects, which, for her, included headaches, general body pains, and continuous bleeding.

Provider perceptions of the method included clinical advantages, particularly for women with heavy bleeding or fibroids, the contraceptive effectiveness of the method, and the ability of the method to reduce menstrual bleeding and cramps.

Providers liked the clinical advantages of the LNG IUS, including its contraceptive effectiveness and its ability to reduce bleeding, cramps, and fibroids.

*One of the advantages just as I've always said is bleeding, you know it has tendency to reduce menstrual flow … then also another advantage it has, especially those that did not take permission from their husband. You know when they do something inside, their husband will not know, I think that is another advantage.* —Provider, mobile outreach, Nasarawa State

All providers said they would continue offering the method. The main reasons providers cited for continuing to offer the LNG IUS were the method's noncontraceptive benefits, fewer side effects compared to other methods, and positive testimonies from clients.

*Number one is the report I have been getting from my clients. I will no more be getting those reports of “Madam, this thing is giving me bleeding” and if your client come to you like those, even those ones that are not coming for family planning, if you send somebody for scan and you are told that she has little, little fibroid, if you know this thing [LNG IUS] can help shrink it, you have solved her problem now. So, I will continue with it. I love this new method, I love it.* —Provider, social franchise, Anambra

Most providers had nothing negative to say about the method, as illustrated by a mobile outreach provider from Abuja: “It doesn't have any disadvantages. Rather it has more advantages.” Perceived disadvantages that were mentioned included the cost, mentioned by 2 providers, and bleeding changes, mentioned by 4 providers. Three providers mentioned expulsions of the method, particularly with their first insertions, as another disadvantage of the method.

#### Perspectives on Method-Related Bleeding Changes Among LNG IUS Clients

Among the 12 women who reported having reduced bleeding, most said they were comfortable with lighter periods. In some cases, women clarified that while they were happy with reduced bleeding, they would have been opposed to amenorrhea. Among the 7 women who reported amenorrhea, 2 were not happy with this change, but all others mentioned they were comfortable with not bleeding. These women noted that they were either counseled about the possibility of amenorrhea or they preferred not bleeding.

*With the method I had before, it comes for 5 days and it will be heavy but since I did this one it is very scanty, like blood stain on pants on the third or fourth day and it will not rush as usual. We have been told that there is no cause for alarm. … I have accepted it. It has no [negative] reaction in the body.* —User, age 32, 4 children

### Qualitative Interviews With Key Opinion Leaders

All of the key opinion leaders reported being familiar with the method before the interview. When asked what method attributes they believed would be most attractive to women, almost all mentioned reduced menstrual bleeding. For example, one key opinion leader said:

*For LNG IUS specifically, I think you also need to highlight the fact that it can play a significant role in reducing menstrual blood flow, which can be such a nuisance for many women.*

Other attributes commonly mentioned included the method's long-acting duration, high effectiveness, noncontraceptive clinical health benefits, and reduced menstrual cramps and pain. One respondent noted:

*Well, the product has its advantages … giving women the choice to make both contraceptive and noncontraceptive choices at the same time—killing 2 birds with 1 stone.*

When asked about disadvantages and barriers to wider use of the method, the most common response was high commodity costs, mentioned by 14 of the 17 key opinion leaders. Other barriers frequently mentioned included a shortage of trained providers and heavy provider workloads, low availability of the method, and the invasiveness of the insertion procedure, especially compared with implants. For example, one key opinion leader said:

*The insertion method through the vagina may not be acceptable to some groups of women, especially where there is the option of the less invasive implants.*

Key opinion leaders acknowledged the advantages of the LNG IUS but also expressed concerns about high commodity costs of currently available products.

The majority of key opinion leaders felt that demand for the LNG IUS would increase if new, more affordable products were introduced. For example, one said, “If it's made more affordable, yes, it will improve access and certainly uptake will increase.” However, several respondents indicated that price could remain a challenge relative to the copper IUD; for example, one respondent said, “The main barrier [with the LNG IUS] is the competition with copper IUD in terms of pricing.” Several key opinion leaders noted that many clients and providers have a preference for implants over copper IUDs, which could also influence demand for the LNG IUS.

*I don't think the reduction in price on its own can cause an increase in demand. I think the preference for implants generally over IUDs or intrauterine systems has much more to do [with other factors] than just the cost because the implants are much more expensive than copper T IUD but they are much more popular if you look at the uptake.*

When asked what segments of the population would be most likely to use the LNG IUS if new, more affordable options were available, the most common response was women who are seeking clinical benefits of the method, with 12 respondents mentioning this group.

*I think the niche for LNG IUS is for treatment of those menstrual abnormalities in addition to contraception.*

Recommended strategies to increase voluntary uptake of the method included health care provider training, demand creation, stakeholder engagement, and commodity security.

*The challenge is making sure that the providers are well trained, the method is available at all times, and the women are aware of it. … I think these are the challenges; once we solve them, there won't be any problem.*

Several key opinion leaders noted the importance of expanding access to the method in both the public and private sectors:

*If you use the total market approach, yes! It will thrive better in the private sector and social marketing sector—where women would want to pay for the services not necessarily where it is free. … And then to get the consensus of the government to include it within the basket of commodities available in the public health facilities, it will help to increase access to it.*

Key opinion leaders also acknowledged the importance of government involvement and support for scale-up of the method.

*It won't be widely available if it is only in the private sector, if it is not one of the products that the Federal Ministry of Health procures for distribution. … Only the government can actually scale up this and make sure that it is widely available.*

## DISCUSSION

Just under 1,000 women in 16 states of Nigeria had an LNG IUS inserted between September 2016 and December 2017 by MSION-supported providers, representing less than 1% of the voluntary LARCs delivered by these providers during the time frame. This outcome was similar to results from a pilot introduction of the ICA Foundation product by Marie Stopes Kenya, which documented low uptake of the LNG IUS compared with other LARCs.^15^ For MSION, it proved challenging to introduce a new method through a service delivery infrastructure focused on expanding access to existing LARCs and that often experienced an overwhelming demand for previously unavailable methods, such as implants, in rural settings. Nigeria is experiencing increased implant use in line with broader trends across sub-Saharan Africa.^16^ The barriers preventing similar uptake of IUD use, such as common misconceptions about the method among both clients and providers,^17^ may be even more acutely relevant for the lesser-known LNG IUS. Several key opinion leaders noted that women and providers may prefer implants over the LNG IUS even if the method were more affordable and widely available, because for women who want a long-acting hormonal method, the implant is better known and is seen as a less invasive procedure. FHI 360 is currently working with partners to conduct additional market research to better understand potential demand for the LNG IUS in Nigeria.

LNG IUS insertions made up less than 1% of all voluntary LARCS delivered during the16-month time frame.

There were also specific supply- and demand-side factors that may have limited LNG IUS uptake. One major challenge was that efforts to generate demand for LNG IUS were limited. Awareness-raising activities were integrated into existing materials and channels, and additional emphasis was not given to the LNG IUS, partly due to staff concerns about appearing to “promote” one method over another. This is reflected in the low proportion of users who had heard about the LNG IUS from community mobilizers or other awareness-raising activities. According to the 2017 MSION client exit interviews, 54.2% of MSION clients knew which method they wanted before coming for the service, demonstrating the importance of community-level awareness raising, especially for a new and lesser-known method such as the LNG IUS. Recommendations from friends and family played an important role for LNG IUS social franchise clients, with almost 40% hearing about the LNG IUS from friends or family, suggesting a valuable role for LNG IUS “satisfied users” in awareness-raising and demand-creation activities.^18^ This indicates that uptake may remain low until there is a critical mass of voluntary early adopters, which will require more demand-side activities and a longer time frame than were possible for this program. Our analysis found some differences between MSION LARC clients and women choosing the LNG IUS, which could be explained in part by the high proportion of implant users among MSION LARC clients, who are typically younger and less educated than IUD users. Further research is planned to assess different characteristics of LNG IUS adopters compared with other family planning users, which could assist with more nuanced demand generation and forecasting efforts.

Challenges related to generating demand for LNG IUS may have limited uptake of the method.

On the supply side, a small pool of providers (40% of mobile outreach teams and 7% of social franchise clinics) were responsible for delivering almost half of all LNG IUS delivered during the program period. Further evaluation is needed to ascertain the extent to which this was due to provider-side factors (e.g., expertise and motivation) or environmental factors (e.g., location and type of clientele served by these providers), but this finding is in line with other similar research, including an assessment of introducing postpartum IUD services (with the copper IUD) in Rwanda, which found that having engaged providers and managers was a critical factor for success.^19^ The important role the provider played in LNG IUS uptake is also reflected in the findings from the qualitative interviews—that women's main reason for choosing the LNG IUS was based on provider recommendation. Similarly, providers were reported as the primary source of information for the LNG IUS for almost 80% of LNG IUS clients; in mobile outreach settings, which serve more rural populations where LNG IUS awareness is likely to be very low, this rose to 99%.

These and other similar findings^20^ suggest that at least in the initial product introduction phase, training and supervision investments should be targeted at those most likely to become “provider champions” who may build momentum around LNG IUS provision and demonstrate the potential of the LNG IUS to their peers as part of a comprehensive approach to voluntary family planning. There is also a need for additional research to further understand providers' perspectives on who they view as appropriate clients for the LNG IUS. Interviewed providers emphasized the clinical benefits of the LNG IUS for women with heavy bleeding or fibroids, and this may lead them to view the LNG IUS as a “niche” service for women presenting with these issues. If this were the case, implementers could consider adapting provider counseling training to emphasize that the LNG IUS can be used by multiple different client groups to meet their contraceptive needs. Future training should also be informed by the qualitative findings from some providers on some initial difficulties with LNG IUS insertion.

Most LNG IUS adopters reported that they would have chosen another LARC if the LNG IUS had not been available (MSION-supported providers are known within Nigeria to be able to provide LARC services, so it is unsurprising that women seeking services from these providers would have a preference for LARCs). However, 20% of LNG IUS adopters indicated that they would have used a short-acting or traditional method, or not used any method, if the LNG IUS had not been available. This is in line with findings from a study of LNG IUS users in Kenya^21^ and suggests that for a sizable minority of women, the addition of the LNG IUS to the family planning service mix may lead them to switch from a less effective, short-acting method of family planning.

The addition of the LNG IUS to the family planning method mix may lead some women to switch from a less effective, short-acting method.

The most cited reason for choosing the LNG IUS in the supplemental LNG IUS client data was the potential of reduced menstrual bleeding (61%). Reduced menstrual bleeding was cited as a reason for use by over half of social franchise LNG IUS clients and almost all (93%) of mobile outreach LNG IUS clients, a notable difference that is worth further investigation. This finding was also reflected in the qualitative interviews, where women frequently mentioned choosing the method because they were told that it could help reduce menstrual bleeding (as well as treat fibroids). Although most users who experienced reduced bleeding or amenorrhea were happy with the change, a small number were not. This finding is supported by Polis and colleagues (2018) who found that women's reactions to contraceptive-induced menstrual bleeding changes can vary widely.^22^ Moving forward, there is a need for additional evidence regarding whether reduced or no bleeding associated with use of the LNG IUS is something that different segments of women perceive as an advantage or disadvantage^23^; for women who would welcome this attribute, it could be emphasized in targeted demand-generation efforts.

Overall, the majority of interviewed LNG IUS users reported positive experiences with the method. A perceived lack of side effects was the most frequently mentioned advantage. This finding is similar to results from qualitative interviews with Mirena users in Kenya, which documented that women's main reason for choosing the LNG IUS as their family planning method was the perception that the method had fewer side effects compared with other contraceptive methods.^24^ Since the most common reason for non-use of contraception among women in developing countries is concern about side effects and health risks,^25^ this is also a product attribute that should be included in counseling and demand-creation efforts.

Several key opinion leaders noted the importance of a total market approach to product introduction, which would require support and engagement from the Federal Ministry of Health as well as a coherent strategy of introduction across the public and private sectors in order to maximize access for women. Most key opinion leaders felt that demand for the method would increase if more affordable products were introduced. However, all LNG IUS services under the MSION program were free or subsidized, and no change in uptake was seen during the 3 months when the price was dropped in social franchises. This suggests that product affordability alone, although an important prerequisite, will not be sufficient to generate voluntary LNG IUS uptake at scale, as discussed elsewhere.^4^

### Limitations

Our study had important limitations. Despite concerted efforts by MSION, the supplemental data specific to LNG IUS clients were collected by providers for only 38% of LNG IUS clients and were systematically missing for certain providers. Sociodemographic data were available for only 30% of clients. Furthermore, provider-collected data may be subject to bias as the user may offer the response they believe the provider wants to hear or may be unwilling to respond negatively.

The target sample size for in-depth interviews of clients at mobile outreach and public-sector sites was not met. As a result, we addressed the general themes around the LNG IUS as developed in our protocol, but we were unable to draw comparisons across different channels. The low number of public-sector clients also meant that we were unable to offer insights on LNG IUS delivery through the public sector—an unfortunate gap because the public sector would be a key delivery channel for any LNG IUS scale-up.

The client sample for both routine data and qualitative data was limited to those who had used the method, so we could not report on the perspectives of women who did not choose the LNG IUS. Similarly, the perspectives of women who had discontinued the method were not well represented in the sample of qualitative interviewees. Finally, the perspectives of the key opinion leaders interviewed may not be representative of other stakeholders' perspectives or adequately predict the potential of the method if it is scaled up in Nigeria.

## CONCLUSION

MSION's experience, along with feedback from the providers, LNG IUS users, and key opinion leaders, suggests that the LNG IUS has attractive benefits for users, but that without adequate demand-generation activities, supportive providers, and satisfied clients to generate interest, initial uptake may remain low. As such, a holistic and multi-stakeholder approach with an affordable product and coordinated demand- and supply-side activities, including cultivating a body of provider and user champions, may be required for this method to reach its full potential in Nigeria and other similar settings.

## Notes

**Peer Reviewed**

**Funding:** This paper was made possible in part by the support from the American People through the United States Agency for International Development (USAID) through the Support for International Family Planning and Health Organizations (SIFPO) 2: Sustainable networks project, Cooperative Agreement No. AID-OAA-A-14-00036, and from the Bill & Melinda Gates Foundation.

© Eva et al. This is an open-access article distributed under the terms of the Creative Commons Attribution 4.0 International License (CC BY 4.0), which permits unrestricted use, distribution, and reproduction in any medium, provided the original author and source are properly cited. To view a copy of the license, visit http://creativecommons.org/licenses/by/4.0/. When linking to this article, please use the following permanent link: https://doi.org/10.9745/GHSP-D-18-00242
